# Some Epidemiological Aspects of Cutaneous Leishmaniasis in a New Focus, Central Iran

**DOI:** 10.1155/2015/286408

**Published:** 2015-09-21

**Authors:** M. R. Yaghoobi-Ershadi, N. Marvi-Moghadam, R. Jafari, A. A. Akhavan, H. Solimani, A. R. Zahrai-Ramazani, M. H. Arandian, A. R. Dehghan-Dehnavi

**Affiliations:** ^1^Department of Medical Entomology and Vector Control, School of Public Health, Tehran University of Medical Sciences, Tehran, Iran; ^2^Isfahan Research Station, National Institute of Health Research, Isfahan, Iran; ^3^Yazd Health Research Station, National Institute of Health Research, Yazd, Iran; ^4^School of Public Health, Yazd University of Medical Sciences, Yazd, Iran

## Abstract

Following the epidemic of cutaneous leishmaniasis in Khatam County, Yazd Province, this study was carried out to determine vector, and animal reservoir host(s) and investigate the human infection during 2005-2006. Four rural districts where the disease had higher prevalence were selected. Sticky paper traps were used to collect sand flies during April to November, biweekly. Meanwhile rodents were captured using Sherman traps from August to November. Households and primary schools were visited and examined for human infection in February 2006. The parasite was detected by RAPD-PCR method. The rate of ulcers and scars among the inhabitants was 4.8% and 9.8%, respectively. Three rodent species were captured during the study: *Meriones libycus, Rhombomys opimus*, and *Tatera indica*. Six sand fly species were also collected and identified; among them *Phlebotomus papatasi* had the highest frequency. *Leishmania major* was detected as the agent of the disease in the area. It was detected from *R. opimus* and native people.

## 1. Introduction

In the old world, cutaneous leishmaniasis (CL) is a major public health problem in the Eastern Mediterranean Region of World Health Organization (WHO), with more than reported cases [1]. In this region, Iran is known as one of the high risk countries for the disease with more than 20000 annual cases. The endemic foci of CL are reported in 17 out of 31 provinces of the country while foci of Isfahan, Yazd, Khorasan-e-Razavi, Golestan, and Fars Province are more important [2–11].

Previous studies in Iran indicated* Phlebotomus papatasi* as the main vector among 45 reported sand fly species [12, 13].* Rhombomys opimus* and* Meriones libycus* are the main and secondary reservoir hosts and* Leishmania major* is the agent of zoonotic cutaneous leishmaniasis (ZCL) [2–11].

In recent years, CL cases have been reported increasingly from different areas of Yazd Province, central Iran, including Khatam County. Due to lake of data in this area, current study was designed and conducted to determine some epidemiological aspects of the statistical evaluation of patients with cutaneous in Health Center of Yazd during 2000–2004 that shows that most of cases exist in Khatam city, the southernmost province of Yazd There were 1509 cases recorded during these years. Most cases are in Herat (Fathabad Village) and Marvast (village of Harabarjan), and it has become a common disease in the last four years.

## 2. Objects and Methods

### 2.1. Study Area

Khatam County with 7931 km^2^ area and total population of about 35000 is located in the southeast of Yazd Province, central Iran. The elevation of the county ranges between 1500 and 3005 m above the sea level. The climate is hot and dry. Khatam County has two towns of Marvast and Harat as well as four rural districts of Fathabad, Harabarjan, Chahak, and Isar. Agriculture, animal husbandry, service affairs, office affairs, and trading are the main occupations in the county (Yazd Province statistics 2005). The study was carried out in Fathabad, Harabarjan, and Marvast areas.

### 2.2. Study of Human Infection

Prevalence of cutaneous leishmaniasis was studied during February and 300 households were visited and interviewed. Family size, infected members, age, gender, disease condition (acute wound or scar), place or number of wounds, and time and place of infection were considered in the questionnaires. Smears were prepared and examined from the acute lesions of native people who had no history of travel to other endemic foci of the disease during past year. All students of the primary schools were also visited. The serosity of lesions was inoculated to the tail base of Balb/C mice in the field. After infection of the mice and wound formation, the serosity of their wounds was cultivated in the Schneider-RPMI media. After one week, promastigote stage of the parasite was developed and used for species identification using RAPD-PCR [14].

### 2.3. Study of Rodents

For this purpose wild rodents were captured around the study area during September to November biweekly using Sherman traps. Traps were installed before sunset and collected next early morning. Trapped rodents were identified using morphological and morphometric characteristics [15] and after anaesthesia two slides were prepared from each ear of the rodents [16]. Species recognition results and direct examination of parasitology were recorded by number, date, and trapping location.

### 2.4. Study of Sand Flies

Sand flies were collected biweekly from indoor (bedrooms, toilets, hall, etc.) and outdoor (rodent burrows) resting places using 30 sticky traps (castor oil coated on A_5_ white papers) from the beginning to the end of the active season. The traps were placed before sunset and collected next early morning. For species identification, sand flies were mounted in Puri's medium and identified using the relevant keys [17]. Sex ratio was calculated as the number of males/females × 100. Study on sand fly infection was performed during August-September using dissection method and direct observation of head and gut under the light microscope. Susceptibility of sand flies to Deltamethrin was evaluated using WHO test kits in the diagnostic dose [1].

### 2.5. Parasite Detection

Schneider medium was used to transfer and culture the parasite in the field. The parasites then were transferred to RPMI medium in the laboratory and, after sufficient amplification, the species was identified using RAPD-PCR method [14].

### 2.6. Statistical Analysis

SPSS version 11.5 and *χ* ^2^ test were used to analyse data.

## 3. Results

### 3.1. Human Infection

During February 2006 a total of 300 families with a population of 1364 (696 males and 668 females) were visited and their demographic as well as the disease data was recorded. Out of them, 4.64% had acute lesions (Table 1). *χ* ^2^ analysis showed no significant difference between males and females (*α* = 5%). Most of the wounds were found in 10–14 years age group (8.1%), while the lowest rate was found in 20–24 years age group (1.72%). All students of primary schools of the area were also visited. The active lesions were found in 4.94% of them (Table 2). There was no significant difference between boys and girls (*P* > 0.05).

The isolated parasite from the local patients with no history of travel was detected as* L. major*.

### 3.2. Rodents

**Figure 1:**
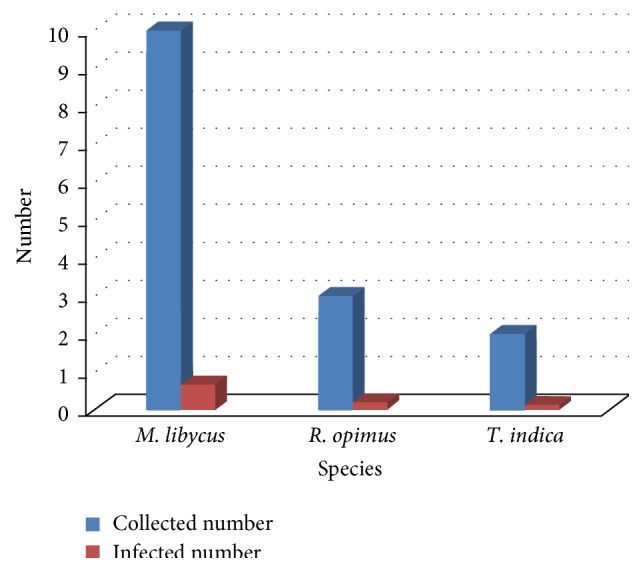
Trapped rodents and their infection to* Leishmania *parasite, Khatam County, Yazd Province, central Iran, 2006.

During this study 15 rodents were trapped and identified as follows:* Meriones libycus* (66.7%),* Rhombomys opimus* (20%), and* Tatera indica* (13.3%). One* M. libycus*, one* T. indica,* and all* R. opimus* specimens were found to be infected with the amastigotes of* Leishmania* parasite (Figure 1). The isolated parasites were identified as* L. major*.

### 3.3. Sand Flies

**Figure 2:**
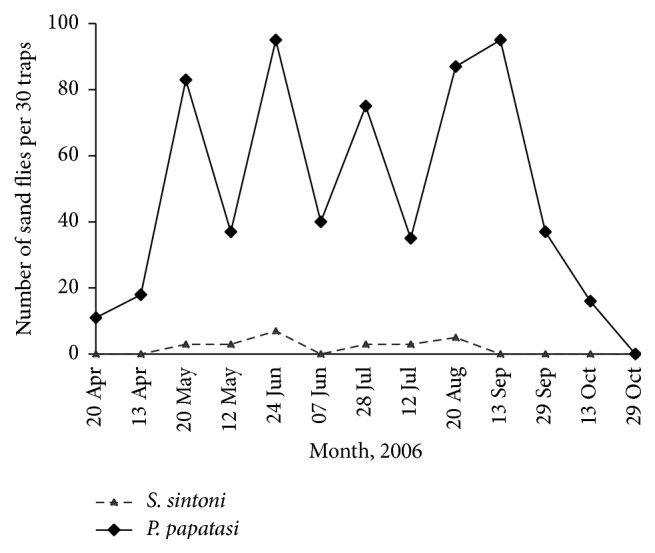
Monthly prevalence of* Se. sintoni, Ph. papatasi* indoors of Khatam County, Yazd Province, central Iran, 2006.

**Figure 3:**
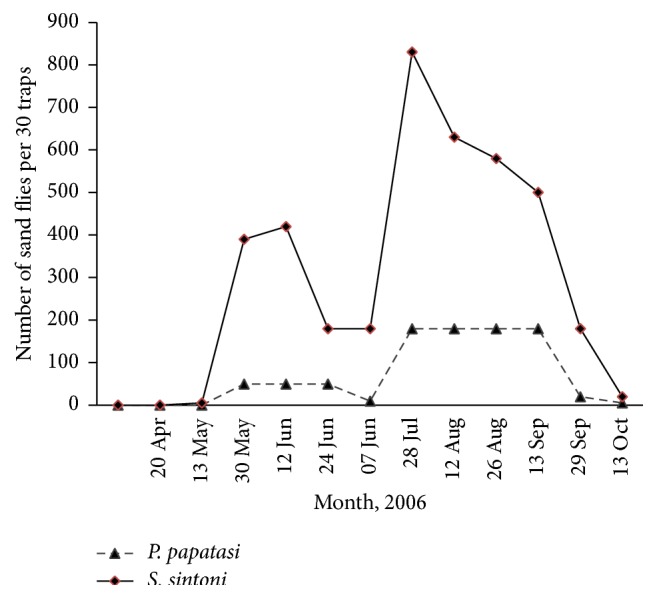
Monthly activity of* Se. sintoni, Ph. papatasi* outdoors of Khatam County, Yazd Province, central Iran, 2006.

A total of 5571 sand flies (772 indoors and 4999 outdoors) were collected and identified as* Phlebotomus papatasi* (85.35%),* Ph. mongoliensis* (0.03%),* Ph. kazeruni *(0.03%),* Ph. sergenti* (0.03%),* Sergentomyia baghdadis* (0.3%), and* Se. sintoni* (14.28%). The dominant species were found to be* Ph. papatasi* and* Se. sintoni*, respectively.* Phlebotomus mongoliensis *and* Ph. sergenti* were collected only indoors, while* Se. baghdadis* was found only outdoors (Table 3). The monthly activity of two main species, that is,* Ph. papatasi* and* Se. sintoni* both indoors and outdoors, was studied. Both species were collected in all sampling months, from May to October with 2 peaks of activity (Figures 2 and 3).

Sex ratio for* Ph. papatasi* was calculated as 383.55 indoors and 159.76 outdoors. This ratio for* Se. sintoni* was found to be 106.25 and 239.48 in indoor and outdoor resting places, respectively.

During August and September a total of 295 female sand flies were dissected for the parasitic infection, but finding the promastigote was failed.

Susceptibility test with Deltamethrin 0.025% resulted in 100% mortality in tested* Ph. papatasi*.

## 4. Discussion

This is the first time that an epidemiological study was conducted in Khatam County. In this survey six sand fly species were collected and identified; among them* Ph. papatasi* was the dominant species indoors (95.2%). This high indoor density confirms the endophilicity behavior of this sand fly and proves its domestic comparing other collected species. It is also found that because of neighborhood of rodent burrows and villages* Se. sintoni* had a relative high density indoors. This is the same as some other parts of Iran [7, 18].

Although the promastigotes of* Leishmania* parasite were not found in dissected* Ph. papatasi* specimens, high density of this species in both indoor and outdoor resting places, as well as isolation of* L. major* from this sand fly in close foci, that is, Ardakan, seems to be the main vectors of cutaneous leishmaniasis in the study area and has a critical role in the disease transmission from rodents to human [19, 20]. Test of* Ph. papatasi* against Deltamethrin 0.05 showed it is susceptible. Although there is a report of tolerance to DDT 4% in this species [21], it is now susceptible to all tested insecticides. Therefore, these insecticides can be used in epidemics of zoonotic cutaneous leishmaniasis for sand fly control [22, 23].

With due attention to leptomonad infection of all three captured rodents, detection of* L. major *from* R. opimus,* and history of studies in Iran,* R. opimus* and* M. libycus* are introduced as reservoir hosts of ZCL in Khatam County. These gerbils have been found to be infected in central and northeastern foci of the disease in Iran [2, 3, 6, 7, 9, 20, 21, 24–27].

Study on prevalence of the disease in the inhabitant population of Khatam County showed the acute lesions existed in all age groups and both genders (*α* = 0.05). This means we encounter a new focus of cutaneous leishmaniasis. As* L. major* was isolated from native people without history of travel, it can be concluded that ZCL has prevalence in the county. This parasite is the causative agent of ZCL in other foci of the disease in Iran [7]. So, new focus of ZCL has been established in Khatam County with* L. major* as agent,* Ph. papatasi* as suspected vector, and* R. opimus* as the main reservoir host. It also seems that* M. libycus* and* T. indica* play a role in preserving this focus with* R. opimus.*

It is conjectured that development of agriculture in the area, especially cultivating crops like sugar beet and vegetables, and construction of numerous water wells for the agricultural purposes have increased the soil moisture and resulted in the colonization of gerbils and increasing the population of sand flies.

Outdoor sleeping behavior of people during the disease transmission months in summer and close contact of farmers with the infected sand flies in farms have resulted in increasing the rate of human infection.

For control of the disease it is suggested to start a rodent control program within a radius of 500 m around the infected/at risk villages prior to starting the active season of sand flies. Health education and improving the knowledge of people regarding ZCL can help to prevent the disease transmission. Self-protection using long-lasting insecticide treated net as well as repellents can also decrease the human-vector contact and therefore collapse the disease prevalence in the area.

## 5. Conclusion

In this study,* P. papatasi and S. sintoni were, respectively, predominant species *both indoors and outdoors.* T. indica, M. libycus, and R. opimus* were found to be infected with amastigotes of* Leishmania *parasite. Study of prevalence among 1364 inhabitants of Marvast and Harat district showed rate of 4.95% for active lesions and the scar rate was 9.75% but our studies showed that the majority of scars were due to the treatment of ulcers in the same year. Based on the findings of this study, zoonotic cycle of the disease exists in the area. Therefore planning for the disease control should be based on this form.

## Figures and Tables

**Table 1:** Prevalence of acute lesion and scar in the studied families, Khatam County, Yazd Province, central Iran, 2006.

Age	Males					Females					Total				
	Visited	Scar		Acute lesion		Visited	Scar		Acute lesion		Visited	Scar		Acute lesion	
		Number	%	Number	%		Number	%	Number	%		Number	%	Number	%
0–4	25	1	4	1	4	28	0	0	1	3.57	53	1	1.88	2	3.7
5–9	45	1	2.22	2	4.44	40	2	5	4	10	85	3	3.52	6	7.05
10–14	72	6	8.33	8	11.11	76	10	1.31	4	5.26	148	16	10.81	12	8.10
15–19	124	16	12.9	5	4.03	134	6	4.47	6	4.47	259	22	8.49	11	4.24
20–24	98	6	6.12	1	1.02	76	7	9.21	2	2.63	174	13	7.47	3	1.72
+25	332	41	17.67	16	4.81	314	37	11.78	15	4.77	645	78	12.09	30	4.65
Total	696	71	10.20	33	4.74	668	62	9.28	32	4.79	1364	133	9.75	65	4.76

**Table 2:** Prevalence of acute lesion and scar in primary schools of the studied area, Khatam County, Yazd Province, central Iran, 2006.

Age	Boys					Girls					Total				
	Visited	Scar		Acute lesion		Visited	Scar		Lesion		Visited	Scar		Acute lesion	
		Number	%	Number	%		Number	%	Number	%		Number	%	Number	%
7	77	3	3.89	2	2.59	71	4	5.63	4	5.63	148	7	9.52	6	8.22
8	72	3	4.16	2	2.77	75	5	6.66	2	2.66	147	8	10.82	4	5.43
9	59	4	6.77	2	3.38	52	3	5.76	1	1.92	111	7	12.53	3	5.3
10	82	16	19.51	6	7.31	75	6	8	6	8	157	22	27.51	12	15.31
11	70	17	10	5	7.14	63	7	11.11	4	6.34	134	14	21.11	9	13.48
12	18	2	11.11	1	5.55	12	1	0.82	0	0	30	13	11.93	1	5.55
Total	379	36	9.94	18	4.74	348	26	7.47	17	4.88	727	62	8.29	36	4.95

**Table 3:** Fauna, density, and sex ratio of sand flies in Khatam County, Yazd Province, 2006.

Species	Indoors		Outdoors		Total		Sex ratio	
	Number	%	Number	%	Number	%	Indoors	Outdoors
*Ph*. *papatasi*	735	95.08	4190	83.82	4925	85.33	383.5	159.76
*Ph*. mongoliensis	2	0.26	0	0	2	0.03	—	—
*Ph*. *kazeruni*	1	0.13	1	0.02	2	0.03	—	—
*Ph*. *sergenti*	2	0.26	0	0	2	0.03	—	—
*Se*. *baghdadis*	0	0	17	0.34	17	0.3	—	—
*Se*. *sintoni*	33	4.27	791	15.82	824	14.28	106.25	239.48
Total	773	13.39	4999	86.61	5772	100	—	—
